# Ion channel traffic jams: the significance of trafficking deficiency in long QT syndrome

**DOI:** 10.1038/s41421-024-00738-0

**Published:** 2025-01-10

**Authors:** Gema Mondéjar-Parreño, Ana I. Moreno-Manuel, Juan Manuel Ruiz-Robles, José Jalife

**Affiliations:** 1 Centro Nacional de Investigaciones Cardiovasculares (CNIC), Madrid, Spain; 2 CIBER de Enfermedades Cardiovasculares (CIBERCV), Madrid, Spain; 3 Departments of Medicine and Molecular and Integrative Physiology, University of Michigan, Ann Arbor, MI USA

**Keywords:** Membrane trafficking, Mechanisms of disease

## Abstract

A well-balanced ion channel trafficking machinery is paramount for the normal electromechanical function of the heart. Ion channel variants and many drugs can alter the cardiac action potential and lead to arrhythmias by interfering with mechanisms like ion channel synthesis, trafficking, gating, permeation, and recycling. A case in point is the Long QT syndrome (LQTS), a highly arrhythmogenic disease characterized by an abnormally prolonged QT interval on ECG produced by variants and drugs that interfere with the action potential. Disruption of ion channel trafficking is one of the main sources of LQTS. We review some molecular pathways and mechanisms involved in cardiac ion channel trafficking. We highlight the importance of *channelosomes* and other macromolecular complexes in helping to maintain normal cardiac electrical function, and the defects that prolong the QT interval as a consequence of variants or the effect of drugs. We examine the concept of “interactome mapping” and illustrate by example the multiple protein–protein interactions an ion channel may undergo throughout its lifetime. We also comment on how mapping the interactomes of the different cardiac ion channels may help advance research into LQTS and other cardiac diseases. Finally, we discuss how using human induced pluripotent stem cell technology to model ion channel trafficking and its defects may help accelerate drug discovery toward preventing life-threatening arrhythmias. Advancements in understanding ion channel trafficking and *channelosome* complexities are needed to find novel therapeutic targets, predict drug interactions, and enhance the overall management and treatment of LQTS patients.

## Introduction

Long QT syndrome (LQTS) is a rare but highly arrhythmogenic cardiac disease characterized by an abnormally prolonged QT interval on the electrocardiogram (ECG)^1^. In the context of a prolonged QT, it is common to observe the appearance of Torsade de pointes (*TdP*) events^1,2^. *TdP* is a life-threatening polymorphic ventricular tachycardia that can degenerate into ventricular fibrillation and sudden cardiac death (SCD)^2^. Sixteen subtypes (LQTS1-16) of heritable LQTS, previously known as Romano-Ward Syndrome, have been associated with monogenic variants in 16 genes with autosomal dominant inheritance (Table 1). Two of these genes (*KCNQ1 and KCNE1*) also cause the rare autosomal recessive form of Jervell Lange-Nielsen Syndrome (JLNS1-2), associated with deafness (Table 1)^3–5^. Four variants altering highly conserved amino acids in the transient potential melastatin 4 (*TRPM4*) gene, were identified in a cohort of 178 LQTS patients^6^. These variants were either exceedingly rare or absent in control populations and no other variants in the major LQTS genes were found in *TRPM4* variant LQTS carriers^6^. Therefore, *TRPM4* might be a new LQTS subtype.

**Table 1:** Subtypes of congenital LQTS and their associated genes, locus, proteins, functional effects, frequency, clinical features, triggers and other diseases.

Subtype	Gene	Locus	Protein	Functional effect	Frequency (%)	Clinical features	Triggers	Other diseases	References
LQT1	*KCNQ1*	11p15.5	KCNQ1 (Kv7.1)	↓*I*_Ks_	40–55	Broad-base T-wave 63% Incidence of Cardiac Events 6%-8% Sudden Death Risk	Exercise, emotion	Short QT syndrome type 2, familial atrial fibrillation type 3	^405–407^
LQT2	*KCNH2*	7q35–36	hERG (Kv11.1)	↓*I*_Kr_	30–45	Bifid T-waves 46% Incidence of Cardiac Events 6%-8% Sudden Death Risk	Auditory stimuli, emotion, exercise, sleep	Short QT syndrome type 1	^155,408^
LQT3	*SCN5A*	3p21–24	Na_v_1.5	↑*I*_Na_	5–10	Long ST, small T 18% Incidence of Cardiac Events 6%-8% Sudden Death Risk	Sleep	Familial atrial fibrillation type 10, Brugada syndrome type 1, dilated cardiomyopathy 1E, heart block nonprogressive and heart block nonprogressive type IA, sick sinus rhythm and familial ventricular fibrillation type 1	^409–412^
LQT4 (ankyrin-B syndrome)	*ANK2*	4q25–27	Ankyrin-B	Multichannel interactions	< 1	Structural heart abnormalities associated with sinus node dysfunction (bradycardia) and atrial fibrillation, and CPVT early and delayed after-depolarizations	Emotional or exertional stress		^151^
LQT5	*KCNE1*	21q22.1	KCNE1 (minK)	↓*I*_Ks_	< 1	Low penetrance of LQT in ECG	Mainly awake at rest		^413^
LQT6	*KCNE2*	21q22.1	KCNE2 (MiRP1)	↓*I*_Kr_	< 1		Exercise	Familial atrial fibrillation type 4	^314,414^
LQT7 (Andersen-Tawil syndrome type 1)	*KCNJ2*	17q23	K_ir_2.1	↓*I*_K1_	< 1	Muscle weakness, periodic paralysis, and facial dysmorphism. Low penetrance of LQT in ECG	Resting after exercise, cold temperatures and little potassium	Familial atrial fibrillation type 9, short QT syndrome type 3	^138,415,416^
LQT8 (Timothy syndrome)	*CACNA1C*	12p13.3	Ca_v_1.2	↑*I*_Ca_	< 1	Hand/foot, facial, and neurodevelopmental features. Cardiac malformations, intermittent immunological deficiency, hypoglycemia, cognitive alterations including autism, interdigital fusion, and prolonged QT,	General anesthesia (mainly sevoflurane)	Brugada syndrome type 3, neurodevelopmental disorder	^417–419^
LQT9	*CAV3*	3p25	Caveolin 3	↑*I*_Na_	< 1	Predominant in black ethnicity. Mild myophatic features		Familial hypertrophic cardiomyopathy, creatine phosphokinase elevated serum, Tateyama type distal myopathy, rippling muscle disease type 2	^18,420,421^
LQT10	*SCN4B*	11q23.3	Na_v_1.5 β4	↑*I*_Na_	< 1	Fetal bradycardia, and 2:1 atrioventricular (AV) block			^177^
LQT11	*AKAP9*	7q21–22	AKAP-9 (yotiao)	↓*I*_Ks_	< 1				^39,179^
LQT12	*SNTA1*	20q11.2	α1-Syntrophin	↑*I*_Na_	< 1	No cardiac or skeletal muscle disease			^182,185^
LQT13	*KCNJ5*	11q24	K_ir_3.4 (GIRK4)	↓*I*_KACh_	< 1	Prominent U waves		Familial hyperaldosteronism type 3	^42,422,423^
LQT14	*CALM1*	14q32.11	Calmodulin	Multichannel interactions	< 1	T-wave alternans and 2:1 atrioventricular block		Catecholaminergic polymorphic ventricular tachycardia type 4	^424,425^
LQT15	*CALM2*	2p21	Calmodulin	Multichannel interactions	< 1	T-wave alternans and 2:1 atrioventricular block			^424^
LQT16	*CALM3*	19q13.32	Calmodulin	Multichannel interactions	< 1	T-wave alternans and 2:1 atrioventricular block		Catecholaminergic polymorphic ventricular tachycardia type 6	^424,426^
JLNS1	*KCNQ1*	11p15.5	KCNQ1 (Kv7.1)	↓*I*_Ks_	< 1	Congenital deafness. high risk for sudden death. Longer QT comparing to heterozygous condition.	Emotion		^427^
JLNS2	*KCNE1*	21q22.1–22.2	KCNE1 (minK)	↓*I*_Ks_	< 1	Congenital deafness. Less risk of sudden death than in JLNS1. Longer QT comparing to heterozygous condition.			^428^

SCD is a complication of heart diseases with devastating consequences on the patient and their family. It is considered a major health problem worldwide^7^. SCD may be defined as unexpected death, usually caused by cardiac arrhythmias, within less than 1 h from the onset of symptoms, and without any prior condition that would seem lethal^8,9^. Ventricular arrhythmias lead to SCD in more than 400,000 cases in the United States alone every year, having a lower incidence in some Mediterranean countries^7,10^. SCD occurrence increases significantly from age 35 to 40 years, but its incidence is particularly high in the acute phase of myocardial infarction and in the presence of heart failure. It can arise even in the absence of structural cardiac abnormalities^11–14^. This is the case for channelopathies, in which SCD is sometimes the first symptom^15^. The impact of cardiac channelopathies is impressive, as they are likely responsible for approximately half the sudden arrhythmic death syndrome (SADS) cases and for at least one out of five sudden infant death syndrome cases (SIDS)^16–18^. Alterations in the currents that flow through ion channels create imbalances and predispose to electrical storms, polymorphic ventricular tachycardia or ventricular fibrillation, that could unfortunately lead to SCD. Inheritable arrhythmogenic disorders are characterized by a low prevalence in the population, but the concern arises from their difficult diagnosis and their potential to trigger severe arrhythmias and SCD. Among SADSs, LQTS, Brugada syndrome and catecholaminergic polymorphic ventricular tachycardia (CPVT) are the most common^19–23^. In some of these patients, sodium, calcium, and potassium dysregulation results in ionic instability, electrical alternans and lethal arrhythmias^24–26^. Studying a cohort of 201 Norwegian SIDS victims, Arnestad et al. have demonstrated that genetic variants in LQTS genes are present in 9.5% of SIDS victims: they were found in *SCN5A* (50%), *KCNQ1* (19%), *KCNH2* (19%), *CAV3* (11%), and *KCNE2* (4%)^27^. More specifically, in ATS (also considered as LQTS7), Mazzanti et al. recently reported a 9.3% incidence of SCD^28^. On the other hand, in an 18-year retrospective study in which more than 33,000 infants were considered, the authors saw that a prolonged QT identified on the ECG on days 3 to 4 of life was associated with a high risk of SIDS^29^.

In the specific case of an ion channel, trafficking is understood as the set of processes encompassing the movement and localization of the channel during its life cycle. It includes the steps from its synthesis, forward movement and anchoring to the plasma membrane, and subsequent retrograde internalization until its recycling or, otherwise, its degradation^30,31^. The lifespan depends on the cardiac ion channels and their degradation occurs in specific subcellular complexes^32–34^. In most LQTS subtypes, the genetic alterations are directly classified as trafficking-deficiency variants^35–38^. Others are associated indirectly with ion channel trafficking defects, either because they affect agents that stabilize the ion channel at the membrane or alter other players involved in the intracellular machinery responsible for its forward/backward trafficking^39–44^. Similarly, certain drugs have the potential to cause acquired LQTS (aLQTS) by impacting channel trafficking and gating^45–48^. Additionally, trafficking deficiency may reduce the response to a drug or increase the risk of arrhythmias despite treatment^49,50^. With all, defects at the level of the complex macromolecular machinery involved in the modulation of an ion channel life cycle, considering its continuous trafficking, targeting anchoring, and recycling at the plasma membrane of a cardiomyocyte, appear to be a major source of channel dysfunction leading to cardiac arrhythmias^51,52^.

Although trafficking defects have been reported for certain ion channel variants and drug effects in heterologous expression systems, the characterization of intracellular trafficking in control and disease conditions is lacking in cardiomyocytes. Therefore, its study in a more physiological environment can give the scientific community key information for enhancing the understanding of LQTS. Here, we review the role of ion channel trafficking deficiency in congenital (cLQTS) and drug-induced LQTS. We start with a brief overview of the recognition, diagnosis, different types, clinical manifestations, and treatment of LQTS about the molecular and ion channel changes associated with the disease. We then appraise what is known about the intracellular machinery responsible for the synthesis, trafficking, localization, and turnover of the ion channels that underlie the cardiac action potential (AP). Thereafter we look at the trafficking defects that may underlie some of the genotype-phenotype correlations in different types of LQTS, followed by a brief review of some of the drugs that have been identified to induce aLQTS or rescue ion channel trafficking deficiency. We highlight the importance of the macromolecular complexes in the ion channel life cycle and stress the need to map the interactome of each of the cardiac ion channels and other proteins involved in LQTS. We submit that mapping the interactomes that govern the different phases of the cardiac AP is an essential tool to continue advancing LQTS research and accelerate biomarker and drug discovery. Finally, the arrival and integration of human induced pluripotent stem cell (iPSC) technology, together with ongoing refinements in omics and high-throughput screening (HTS), holds promise for developing next-generation treatments that are more effective and safer in alleviating the ion channel trafficking defects underlying each of the relevant LQTS subtypes.

### LQTS overview

**Fig. 1:**
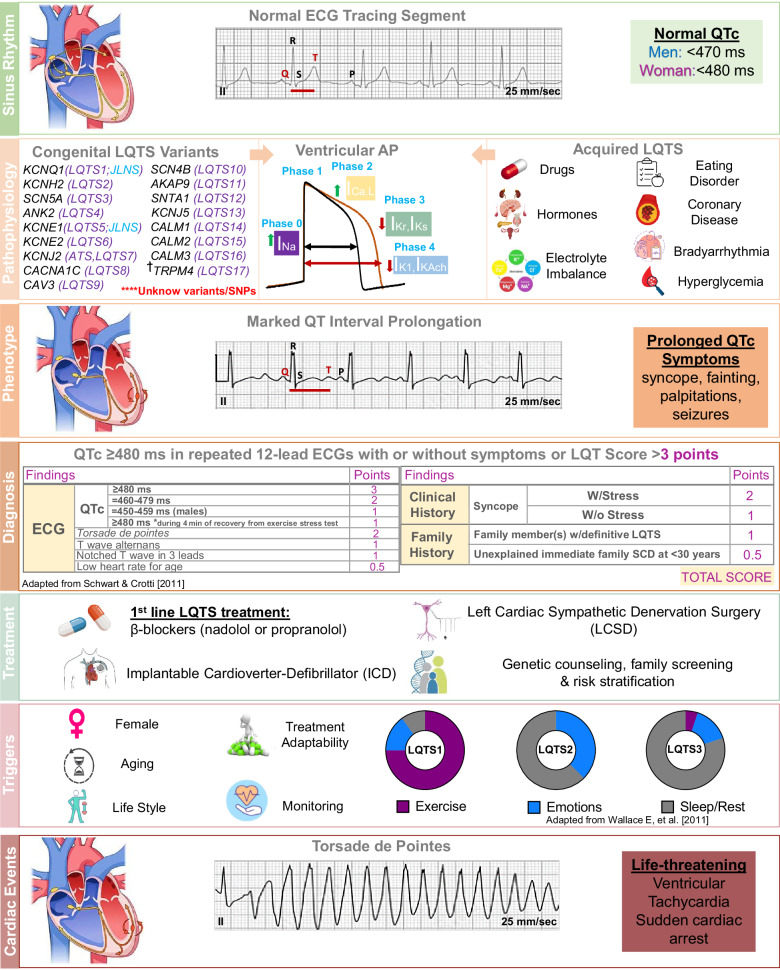
Long QT Syndrome (LQTS) Overview. Illustrative ECGs adapted from public reservoirs JLNS, Jervell Lange-Nielsen; ATS, Andersen-Tawil Syndrome; TS, Timothy Syndrome. Genes related to the autosomal dominant Romano-Ward Syndrome are marked in purple and genes associated with the autosomal recessive form Jervell Lange-Nielsen (JLNS) are marked in blue. ϯ, suspected LQTS–related gene.

The normal QT interval depends on age, gender, and other physiological conditions, but a QT interval ≥ 470–480 ms, in men and women respectively, is sufficient to suspect LQTS^3^ (Fig. 1). Whereas aLQTS may be due to drugs, electrolyte abnormalities, eating disorders, coronary artery disease, bradyarrhythmia, etc., cLQTS is due to genetic variants (listed in Fig. 1) affecting cardiac ionic currents or other proteins that play key roles in controlling the cardiac AP^53^. The three most common subtypes, LQT1 (*KCNQ1*), LQT2 (*KCNH2*), and LQT3 (*SCN5A*) account for 80%-90% of all cases^1^. The remaining heritability has been reported in multiple new minor LQTS genes collectively accounting for 5%-10% of LQTS cases^1,3^. Strong evidence links genes like *CALM1*, *CALM2*, and *CALM3*, coding for Calmodulin, to atypical forms of LQTS often manifesting in infancy^54–57^. *KCNE1* and *KCNE2* variants (encoding mutated versions of minK proteins), although not strongly linked to typical LQTS, show compelling evidence for their association with aLQTS^41,58^. These variants in *KCNE1* may contribute to a milder form of the disease, often requiring a secondary factor to trigger symptoms^59,60^. So, despite causing AP prolongation, each LQTS subtype, and each specific variants represent different underlying mechanisms. Unlike other channelopathies, cLQTS is more unrecognized than rare. Its incidence and prevalence may be higher in certain populations and regions with a higher prevalence of specific LQTS-causing genetic variants^61,62^. However, genotype–phenotype relationships in cLQTS are multifaceted, influenced by specific genetic variants, genetic heterogeneity, incomplete penetrance, variable expressivity, and environmental and triggering factors, all important factors that render disease diagnosis difficult^63^. The primary symptoms in patients with LQTS include palpitations, syncope, and seizures^1,3,64,65^. Interestingly, LQTS accompanied by seizure is often misdiagnosed as primary epilepsy^66,67^. This can occur when a syncopal event (fainting) in LQTS is mistaken for a seizure because both can present with similar clinical features like convulsions^68^. Seizures are often secondary to the cardiac event rather than primary neurological epilepsy^69^. Misdiagnosis and improper management of LQTS patients are often likely to result in fatality^70^, since certain anti-epileptic drugs can trigger cardiac abnormalities and should be avoided in those patients^71^. Patients with LQTS do have a higher incidence of epilepsy^72^. Hence, treatment strategies need to be tailored to address both conditions if they coexist^73,74^. LQTS and epilepsy are two distinct medical conditions, but there is evidence suggesting a potential link between them^75^ due to a genetic overlap between LQTS and certain forms of epilepsy^76^. Variants in genes encoding ion channels, which are crucial for normal electrical activity in both heart and brain, can be implicated in both conditions^76^. As stated above, variants in KCNQ1, KCNH2, and SCN5A are primarily associated with LQTS^1^. Similarly, variants in *SCN1A*, *SCN2A* and *KCNQ2* genes are identified as major contributors to epilepsy^77^. However, variants in *KCNQ1, KCNH2, SCN5A, CACNA1C*, and *ANK2* genes have been associated with both LQTS and epilepsy^78–82^. Therefore, defects for ion channels in neurons could lead to neurological disease as well as LQTS^83^. The electrical disturbances caused by ion channel dysfunctions can manifest differently depending on whether they predominantly affect cardiac or neuronal cells^84^. Importantly, both LQTS and epilepsy are associated with an increased risk of sudden unexpected death^85^. SCD in LQTS can be mistaken for sudden unexpected death in epilepsy (SUDEP)^86^. Understanding this connection can improve screening, diagnosis, management, and outcomes for patients presenting with symptoms of either or both conditions.

The diagnosis of LQTS is established by either a QT interval corrected (QTc) for heart rate (HR) ≥ 480 ms in repeated 12-lead ECGs, with or without symptoms, or by an LQTS diagnostic score > 3 (Fig. 1)^87,88^. LQTS diagnosis score is typically calculated considering QTc, other ECG irregularities, the clinical history of syncope, and the family history^89^, as described in Fig. 1. LQTS treatment options are mostly symptomatic, trying to prevent life-threatening arrhythmias (LTA) and minimize the risk of SCD (Fig. 1)^87^. β-blockers, the first line LQTS treatment, reduce the HR and decrease adrenaline sensitivity allowing for more efficient repolarization^89^. For individuals at high risk for LTA, the implantable cardioverter-defibrillator and left cardiac sympathetic denervation surgery may be considered^3^. Gender, aging, lifestyle and others are risk factors for arrhythmic events associated with LQTS^63^. In this sense, different subtypes of LQTS show specific triggers (medications, electrolyte imbalances, emotional stress, and strenuous physical activities) and may respond differently to therapy options^90^. For example, physical exercise is the primary trigger for LTA or cardiac arrest in LQTS1, arousal, and emotional stress are potential triggers in LQTS2, while cardiac events in LQT3 can often occur during periods of rest or sleep (Fig. 1)^91^.

### Ion channel life cycle in cardiomyocytes

The term “trafficking machinery” refers to the intricate intracellular processes that continuously move and recycle electrogenic proteins like ion channels, pumps, and exchangers to specific areas of the cardiomyocyte^51,92^. These areas, known as microdomains (being the t-tubules, lateral membrane, and intercalated discs (ICD) in the sarcolemma; or referring to specific cytosolic organelles), have distinct roles in processes like AP generation, excitation-contraction (e-c) coupling, electromechanical coupling, mechano-transduction, and cell-to-cell communication^93–95^. Such a highly organized structure is essential for the functional polarization of cardiomyocytes and the coordinated transmission of electrical and mechanical signals throughout the heart^94–99^. Specifically, ion channel trafficking control plays a crucial role in maintaining the electro-chemical balance within and among cardiomyocytes^100^. However, the proper functional expression of ion channels can be disrupted at several points including the transcriptional, translational, and post-translational levels^101^. Therefore, understanding and being able to manipulate ion channel trafficking in cardiomyocytes is crucial for the development of therapeutic interventions for various cardiac conditions, particularly arrhythmogenic diseases like LQTS.

**Fig. 2:**
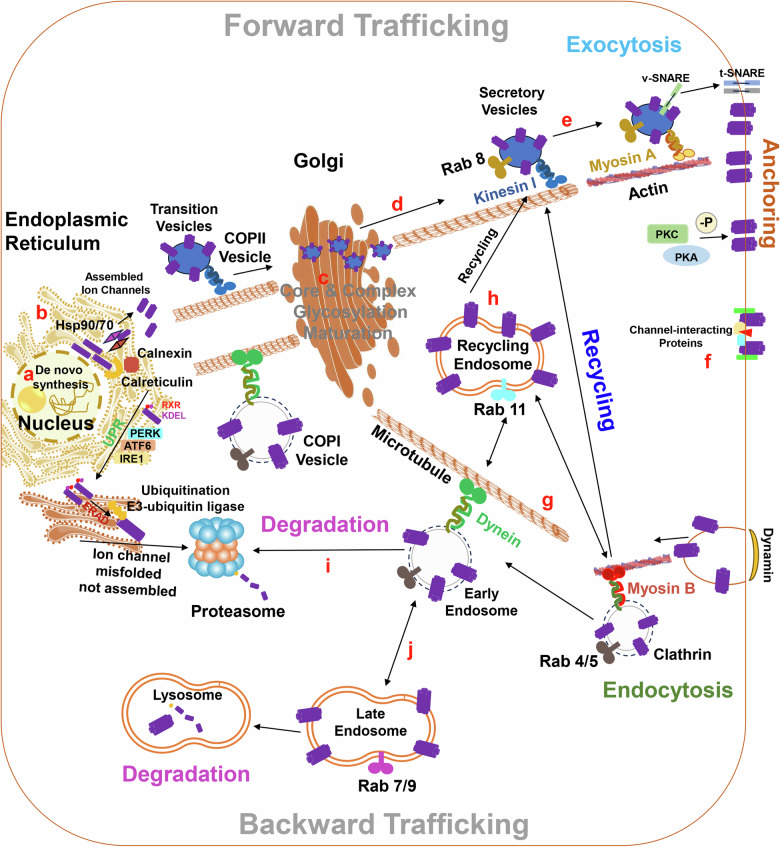
Regulatory steps in the trafficking of cardiac ion channels. **a** Gene transcription. **b** Protein folding and proper assembly. **c** Post-translational modification & glycosylation. **d** Anterograde vesicular trafficking along microtubule. **e** Membrane insertion. **f** Interactions with anchoring, scaffolding and ancillary proteins. **g** Retrograde vesicular trafficking along microtubule (internalization). **h** Recycling. **i** Proteasomal degradation. **j** Lysosomal degradation.

The density of active ion channels in specific membrane domains is a consequence of a dynamic process resulting from the simultaneous and antagonistic action of anterograde (exocytosis, recycling) and retrograde (internalization, degradation) pathways^51^. These processes and the fundamental mechanisms responsible for ion channel trafficking in cardiomyocytes are illustrated in Fig. 2. They may be summarized as follows: Ion channels, much like secreted proteins, are produced within the endoplasmic reticulum (ER). Calnexin, calreticulin and some heat shock proteins (HSPs) assist in the proper folding of newly synthesized ion channels^102–104^. Protein disulfide isomerases (PDI) catalyze the formation and rearrangement of disulfide bonds during ion channel folding^105^. The ER enforces quality control to allow only properly folded channels to leave the compartment^106,107^. Misfolded or incorrectly assembled channels exhibit ER-retention signals, such as the RXR and KDEL motifs, triggering the unfolded protein response (UPR) through three primary pathways: PERK, ATF6, and IRE1. ATF6 and IRE1 activate the transcription of specific target genes related to the ER-associated degradation system, which induces proteasome degradation after ubiquitylation by ER-bound E3-ubiquitin ligases^108^. When correctly folded, ion channels are transported to the Golgi complex (GC) where further processing, like complex glycosylation and other post-translational modifications, occurs^109^. The transport of channels between the ER and the GC occurs via coated transition vesicles^110^. COPII-coated vesicles move toward the GC, and COPI-coated vesicles return to the ER^110^. After leaving the ER or the GC, channels may travel via vesicles until they reach specific cell membranes^111^. Using specific inhibitors of the anterograde trafficking pathways, like Brefeldin A, over the last years researchers have identified unconventional routes for the ion channels movement from the ER to the cell membranes. For example, Kir2.1 and Na_V_1.5 could get to the sarcolemma following GRASP-dependent unconventional traffic pathways^112^. In this way, ion channels essential for maintaining cardiac excitability could be partially positioned and work in the presence of classic trafficking pathway disturbances.

The progression of vesicles transporting ion channels through the cell is facilitated by the presence of molecular motors supporting transport along the cytoskeleton in opposite directions^52,95,113–116^. In the anterograde direction, vesicles travel along microtubules through various kinesins and then along the actin cytoskeleton through myosin Va^117,118^. In the cytoplasm, the coordination of ion channel trafficking also involves various Rab proteins, and monomeric GTPases of the Ras superfamily, which function as markers associated with specific vesicles for intracellular trafficking progression in anterograde and retrograde directions. Rab8 is engaged in the delivery of newly synthesized channels and associated with the secretory vesicle^92^. Once in the destination, anchoring is facilitated by the fusion of a donor compartment (vesicle) with an acceptor compartment (membrane). Each fusion step involves SNARE (an acronym from soluble N-ethylmaleimide-sensitive factor activating protein receptor) proteins carried by both the donor vesicle and the acceptor membrane, destabilizing lipid bilayers and allowing the channel to move to the acceptor compartment during interaction^119^. Ankyrins and spectrin are scaffolding proteins that anchor ion channels to the cytoskeleton and stabilize their insertion and position in the membrane^120^. In these membranous destinations, protein kinases (e.g., PKA and PKC) can modulate ion channel function and trafficking through phosphorylation^121^.

Focusing on the retrograde trafficking from the cell surface, channels become internalized and can either be recycled or degraded in proteasomes or lysosomes. Clathrin and dynamin participate in the endocytosis of ion channels from the membrane^122,123^. In the retrograde direction, the molecular motors include dynein (mostly dynein I) and myosin Vb, associated with microtubules and actin, respectively (Fig. 2)^117,124^. Rab4, Rab5, Rab7, Rab9, and Rab11 are linked to vesicles following ion channel internalization^125–128^. Rab4 and Rab5 are associated with the early endosome, contributing to fast recycling back to the plasma membrane^129^. Rab11 is associated with the recycling endosome, crucial for a slower recycling pathway^130^. Rab7 and Rab9 are associated with the late endosome, directing trafficking proteins toward the lysosome or the proteasome for degradation^52^.

A fine balance between anterograde and retrograde trafficking pathways is necessary for normal cardiac electrical activity. Dysregulation of this balance can be arrhythmogenic. For instance, hypokalemia decreases extracellular K^+^ and triggers hERG channel ubiquitination, internalization, and degradation^131^; hyperglycemia interrupts the interaction between hERG and the chaperone HSP90 and prevents its anterograde trafficking, activating the UPR and channel degradation^132^; cholesterol depletion activates recycling of Kv1.5 from the recycling endosome^133^; and shear-stress or hemodynamic overload also potentiates anterograde ion trafficking^134^.

### Trafficking-deficient variants linked to LQTS

In most LQTS subtypes, we can find genetic alterations classified as trafficking-deficient variants^36,40,43,44,58,59,135–142^. However, the complete repercussions of this type of alterations are unknown, since the percentage of trafficking-deficient variants causing each LQTS subtype is also unknown. Importantly, many of these variants are trafficking defective, yet their biophysical properties remain intact. Here we define loss-of-function as the reduction in channel activity, referring to its action on APD. Therefore, a channel responsible for repolarization (potassium channels) would undergo loss-of-function if the APD is lengthened and causes LQT. In contrast, a channel that opposes repolarization (sodium channel and calcium channels), would experience gain-of-function affecting variants that prolongs APD and LQTS. However, this is a necessary simplification to address all the impairments in LQTS. There are increasing reasons to nuance the classification of variants beyond solely on gain or loss-of-function. Our knowledge of molecular mechanisms is increasing, which has resulted in a growing complexity of the known effects caused by each variant. Below we review the underlying mechanisms of LQTS subtypes centering the attention on the trafficking-deficient variants identified in each case:

- Loss-of-function *KCNQ1* variants linked to LQTS1 can be classified according to their pathophysiological mechanisms: variants can disturb ion permeation, gating, trafficking, Kv7.1-KCNE1 interaction, PKA-mediated signaling, phosphatidylinositol biphosphate binding, and calmodulin binding^140–142^. Trafficking disruption is one of the main pathological mechanisms causing LQTS1. Multiple mechanisms underlying these trafficking deficiencies caused by variants in the *KCNQ1* gene have been described. Among these are misfolding of the Kv7.1 protein, defective ER and Golgi export signals, impaired assembly and stability, altered interactions with chaperone proteins, increased degradation, disruption of post-translational modifications (glycosylation and phosphorylation), and interference with the trafficking machinery^143,144^. The majority of these mechanisms lead to lesser availability of membrane Kv7.1 channels, decreasing the repolarizing K^+^ current (I_Ks_), thus prolonging APD and the QT interval on ECG.
- Most LQT2-linked genetic alterations are missense variants and functional studies suggest that ~88% of them disrupt intracellular trafficking of Kv11.1 gene products to the cell membrane^135^. Similar common mechanisms of ion channel trafficking deficiency caused by variants in the *KCNH2* gene have been described^37,135,145^. Reduction in channel density at the plasma membrane leads directly to decreased I_Kr_, which directly prolongs APD and manifests as a longer QT interval^146^. Membrane reduction in hERG channels responsible for repolarization leads to I_Kr_ loss-of-function, prolonging AP repolarization, which correlates with LQTS.
- On the contrary, the mechanisms responsible for the LQT3-associated phenotype are strongly linked to a gain of function of the α subunit of the cardiac Na^+^ channel (Na_V_1.5) responsible for I_Na_. This current controls phase 0 of rapid AP depolarization, so gain-of-function *SCNA5* variants are associated with increased APD and LQTS^36^. Mechanisms include channel bursting, late reopening, cAMP-dependence, window current, and nonequilibrium gating^147^. However, evidence indicates an interplay between protein trafficking defects and gating abnormalities in the novel Na_V_1.5 F1473S variant causing LQTS3^36^. The response to LQTS3 treatment is influenced by defects in the biophysical properties caused by this specific mutation^36^. On the other hand, the Na_V_1.5 Q1475P variant reduces channel surface expression and I_Na_ density, characteristic of a trafficking defect. The latter variant also leads to positive shifts in the voltage dependence of steady-state activation and inactivation, faster inactivation and recovery from inactivation, and increases the “late” Na^+^ current altering the pharmacological response of the channel^136^.
- Ankyrin-B is now recognized to play essential roles in targeting and membrane stabilization of ion channels, transporters, and signaling molecules such as the sodium-calcium exchanger (NCX1), Na^+^/K^+^-ATPase, and inositol 1,4,5-trisphosphate receptors (IP3Rs)^148–150^. Ankyrin-B variants can lead to mislocalization or dysfunction of these proteins, resulting in abnormal intracellular calcium and sodium handling^148^. The cumulative effect of disrupted ion channel and transporter localization and function can alter the overall excitability of the cardiac cell membrane. Altered excitability can lead to prolonged repolarization and increased susceptibility to arrhythmias^40,148,151^. Most LQTS4 variants are located at or near the regulatory domain of ankyrin-B. Variants in ankyrin-B are difficult to classify as gain or loss-of-function, as they depend specifically on the overall effect on the presence and role of ion channels in the membrane.
- Loss-of-function variants in *KCNE1* linked to LQTS5 can lead to defects in gating, trafficking, Kv7.1-KCNE1 interaction, and adrenergic stimulation^58,59,152^. Variants in KCNE1 can cause misfolding of the protein, which in turn can prevent the proper folding and assembly of the Kv7.1–KCNE1 complex^59,153^. This can lead to mislocalization of the channels within the cell, preventing them from reaching the plasma membrane, and affecting their functional properties, such as gating kinetics and ion selectivity, which can lead to ineffective channel function and contribute to arrhythmic conditions^152,154^.
- The mechanism causing LQTS6 due to *KCNE2* variants is related to altered I_Kr_ gating. However, *KCNE2* variants may also alter hERG channel trafficking^41,155–157^. KCNE2 can form complexes with hERG channels. Improper KCNE2–hERG interactions may result in retention of hERG channels in the ER due to inappropriate folding or assembly, influencing their glycosylation status and reducing the number of functional channels available at membrane level^41^. The complexity of KCNE2 interactions with a variety of K^+^ channels may inform the differential phenotypes observed clinically in patients harboring a *KCNE2* variant^41,137^, reinforcing the idea of not understanding the ion channels as individual or isolated entities.
- As occurs with LQTS1, *KCNJ2* variants related to Andersen-Tawil Syndrome type 1 (ATS1) can be classified depending on their pathophysiological mechanism. Among the more than 90 variants identified for the *KCNJ2* coding protein Kir2.1, ~11% are classified as membrane trafficking variants^138^. Interestingly, ATS1-associated LQTS is increasingly in doubt, because the increment in the LQT interval in these patients is relatively small. Several mechanisms underlying these trafficking deficiencies lead to loss-of-function of Kir2.1, among them: channel protein misfolding; defective ER export signals, impaired assembly and stability, altered interactions with chaperone proteins, increased degradation, disruption of post-translational modifications (glycosylation and phosphorylation), and interference with the trafficking machinery^138^.
- Timothy syndrome is caused by gain-of-function variants in the *CACNA1C* gene, which encodes the Cav1.2 α1_C_ subunit responsible for the depolarizing L-type Ca^2+^ current^158–160^. These variants may disrupt channel inactivation. Interestingly, the *CACNA1C* p.R518C variant exhibits both loss‐of‐function and gain‐of‐function properties^43^. L-type Ca^2+^ channels coded by *CACNA1C* p.R518C exhibit a reduction in current density secondary to a trafficking defect that impedes cell surface expression^43^. The p.R518C mutation in *CACNA1C* changes an arginine (R) to a cysteine (C) at position 518 in the α1C subunit. This amino acid substitution can cause protein misfolding detected by the cellular quality control machinery^161^. The p.R518C mutant of the α1C subunit is likely degraded more rapidly. Retention in the ER and subsequent degradation of the mutant α1C subunit causes a significant reduction in the number of functional Ca_V_1.2 channels that are trafficked to the plasma membrane^161,162^. As in the case of *SCN5A* variants, treatments aimed at improving channel trafficking could have adverse pro-arrhythmogenic consequences due to the multitude of variant effects. Therefore, restoring abnormal trafficking may not be sufficient to correct LQTS in all cases.
- Lipid rafts are specialized, dynamic microdomains in the plasma membrane of cells, enriched with cholesterol, sphingolipids, and certain proteins^163^. Caveolae are a subtype of lipid rafts, characterized by their small flask-shaped invaginations in the plasma membrane^164^. They are particularly rich in caveolin, which is essential for their formation and function^165^. These rafts serve as organizing centers for the assembly of signaling molecules, influencing membrane fluidity and protein trafficking. Lipid rafts and caveolae play significant roles in ion channel trafficking organizing and concentrate specific cardiac ion channels and signaling molecules, facilitating efficient signal transduction and transporting of ion channels, ensuring proper localization and function^166–168^. Caveolae, in particular, assist in endocytosis and recycling of ion channels, maintaining their appropriate surface levels, protecting them from degradation and ensuring their functional integrity^169,170^. They also play a role in the regulation of ion channel activity by facilitating interactions with regulatory proteins and lipids^164,171,172^. Cav-3 is the most abundant caveolin isoform in cardiac tissue. It is responsible for forming lipid raft microdomains and caveolae and potentially plays a role in t-tubule organization^173,174^. Variants in the Cav3-encoding gene are linked to LQTS9^175^. Nav1.5 colocalizes with Cav-3 in lipid rafts, facilitating its stabilization at the membrane and increasing peak I_Na_ density in response to α-adrenergic stimulation^18,175,176^. This, among other reasons, is why variants in Cav3 can disrupt the normal electrical behavior of ion channels.
- Variants in the *SCN4B* gene encoding the Nav1.5 β4 subunit give rise to LQT10^177^. The β4 subunit is an auxiliary protein that modulates the function of the Na_v_1.5 α subunit. These variants alter the biophysical properties of Nav1.5 current and have not been reported to cause any trafficking deficiency, leading to a gain-of-function effect. This can include changes in the activation and inactivation kinetics of the channel. Specifically, gain-of-function mutations may cause the Na_v_1.5 channels to activate more readily or inactivate more slowly, resulting in prolonged sodium influx during the AP. The gain-of-function effect of *SCN4B* variants is also due to the increased persistent sodium current. This persistent current is a small but steady influx of sodium ions during the plateau phase of the AP. It contributes to the overall depolarization and prolongs the APD^177,178^.
- AKAP-9 is a scaffolding protein and a part of the I_Ks_ macromolecular complex. It is critical in compartmentalizing the adrenergically stimulated PKA signaling pathway that leads to I_Ks_ upregulation^39^. No trafficking defects related to AKAP-9 variants have been reported to date. Variants in AKAP-9 produce loss-of-function in I_Ks_ and, therefore, an increase in APD and consequent LQTS, although there is still controversy about the association of this gene with LQTS^179–181^.
- α1-syntrophin (SNTA1), a scaffolding protein, interacts with Na_V_1.5 and Kir2.1 channels via PDZ binding and helps to maintain the normal function of both channels at the lateral membrane^182,183^. Some variants in the α1-syntrophin gene (*SNTA1*) lead to I_Na_ gain of function through several mechanisms and have been classified as LQTS12^44^. Nitric oxide *(*NO) is known to modulate Na_v_1.5 channel activity by inducing nitrosylation, which can reduce sodium current. Variants in *SNTA1* might disrupt this interaction, leading to a reduction in NO production and decreased nitrosylation of Na_v_1.5 channels. This results in an increased sodium current^184^. *SNTA1* variants can directly enhance the conductance of Na_v_1.5 channels and alter the gating properties, making them activate more readily or inactivate more slowly, leading to a larger I_Na_. This is a direct gain-of-function effect on the channel’s biophysical properties^44^. α1-syntrophin is part of larger macromolecular complexes that include various regulatory proteins. Variants in *SNTA1* might disrupt these complexes, leading to a loss of normal inhibitory regulation and an increase in Na_v_1.5 channel activity. Some *SNTA1* variants may specifically increase the persistent sodium current (late I_Na_). This is a small, sustained current that flows during the plateau phase of the cardiac AP. An increase in late I_Na_ can prolong the APD, contributing to arrhythmogenic conditions^182,185,186^.
- Variants in the *KCNJ5* gene, encoding Kir3.4 (or GIRK4), cause LQTS13. Kir3.4 and another GIRK isoform Kir3.1 (GIRK1) may form homo- and heterotetramers to generate the acetylcholine-activated inward rectifying K^+^ current (I_KACh_) current in the heart^187^. The identified G387R variant is within the C-terminus of the Kir3.4 protein, within the region that was proven essential for forming the Kir3.4/Kir3.1 hetero-tetramer^188^. The G387R variant has been shown to reduce the trafficking of Kir3.4 and Kir3.1 to the plasma membrane and decrease I_KACh_ amplitude, producing a loss-of-function responsible for LQTS^42^.
- Calmodulin (CaM) modulates L-type Ca^2+^ and Nav1.5 channels, but also trafficking and assembly of Kv7.1. CaM, acting as a chaperone, binds directly to the C-terminal region of the Kv7.1 subunits, aiding in the assembly of functional tetrameric channel^189^. This interaction is crucial for the channel protein’s proper folding and stability, protecting it from proteasomal degradation^189^. Without CaM, the Kv7.1 channel is prone to misfolding and degradation. Intracellular Ca^2+^ can modulate its interaction with Kv7.1. When calcium levels rise, CaM undergoes a conformational change that can influence the function and localization of Kv7.1 channels^189^. Variants in CAM1-3 coding genes related to LQTS14-16 may reduce Ca^2+^ affinity leading to abnormal Na^+^ and K^+^ currents, and defective intracellular calcium dynamics in the heart^141,142,190^.

### Drugs altering the trafficking of cardiac ion channel related to LQTS

Multiple drugs have the potential to cause arrhythmias by impacting the function of cardiac ion channels through distinct mechanisms; i.e., modifying channel trafficking, directly blocking channel conductance, or both^191^. Disruption of ion channel trafficking may decrease the rate of incorporation or increase the rate of endocytosis of mature channels into the cell membrane, leading to their recycling or degradation^192–194^. Whereas most drugs induce ion channel trafficking defects at therapeutically relevant concentrations, others do so only at toxic doses. Moreover, as many drugs are unspecific, they can impact multiple ion channels, leading to beneficial or harmful out-of-target outcomes^195^. In this sense, considering the ion channels and their regulatory proteins in macromolecular complexes, the therapeutic disturbance of one of them could have relevant consequences on others. The likelihood of causing arrhythmias is not solely dependent on the extent of inhibiting a single ion current. Drugs that mildly inhibit multiple currents may produce similar effects^196^. Nevertheless, discerning the subtle activator/inhibitory impacts on each channel, particularly those associated with ion channel trafficking, poses a challenge in contemporary cardiac safety pharmacology practices^30,191,194^. Pro-arrhythmic effects may not only arise from adverse trafficking and channel block. For example, it is well known that certain classes of chemical compounds, like antidepressants and antipsychotics, are particularly prone to induce channel trafficking defects via similar mechanisms, leading to consequences at the heart level^197,198^. In this sense, the antidepressant tricyclic drug Desipramine, which prolongs the QT interval and induces *TdP*, has been suggested to do so not only via chronic disruption of hERG trafficking but also by direct hERG channel block, acute reduction of hERG surface expression, and induction of apoptosis^199^. Also, the cumulative and often subtle effects of drugs targeting multiple ion channels (due to their absence of specificity and the behavior of channels in macromolecular complexes) can explain why some drugs, even without producing obvious trafficking defects at clinically relevant concentrations, are linked to QT prolongation and cardiac arrhythmias^191^. Although drug-induced trafficking defects in different cardiac ion channels like hERG, Nav1.5, Kv4.3, Kir2.1, GIRK, Kv1.5 and Kv7.1 have been reported, hERG defects are potentially the most dangerous^102,132,191–193,199^. Therefore, drug-induced arrhythmogenic liability during drug development is evaluated based on the nonclinical ICH S7B and clinical E14 guidelines of QT/QTc Interval Prolongation and Proarrhythmic Potential^200^. The focus of such guidelines is on in-vitro inhibition assays of hERG current and in-vivo QTc interval to estimate the risk of QTc prolongation and *TdP* in humans^47^. There are several mechanisms by which drugs interfere in wildtype or mutant ion channel trafficking:

- Direct channel interaction: Some drugs can induce defects in channel trafficking either by directly binding to conserved/canonical drug binding sites for pore blockade and internalization or through other binding sites within the channel structure^201^. A wide variety of structurally diverse drugs are dual inhibitors with equivalent or greater potency for disturbing trafficking compared to direct channel blockade. Examples of dual hERG blockers (Table 2) include the tricyclic antidepressant Desipramine, the selective serotonin reuptake inhibitor Fluoxetine, the macrolide antibiotic Roxithromycin, and the azole antifungal Fluconazole^145,202–204^. Certain ion channel blockers may assist in the proper folding and trafficking of the mutant proteins to the cell membrane, enhancing their stability and functional expression on the cell surface. For instance, high-affinity HERG channel-blocking drugs like E-4031, astemizole, cisapride, and dofetilide (Table 2) can rescue misprocessed hERG channels associated with LQTS2 by stabilizing its conformation, preventing misfolding and subsequent degradation^205–208^. This stabilization can increase the number of functional channels present on the cell membrane. However, these experiments showed that the mutant channel protein could be rescued pharmacologically and raised this as a possible new therapeutic approach. However, pharmacological rescue with high-affinity hERG channel-blocking drugs occurred at concentrations causing complete block of their activity.

**Table 2:**
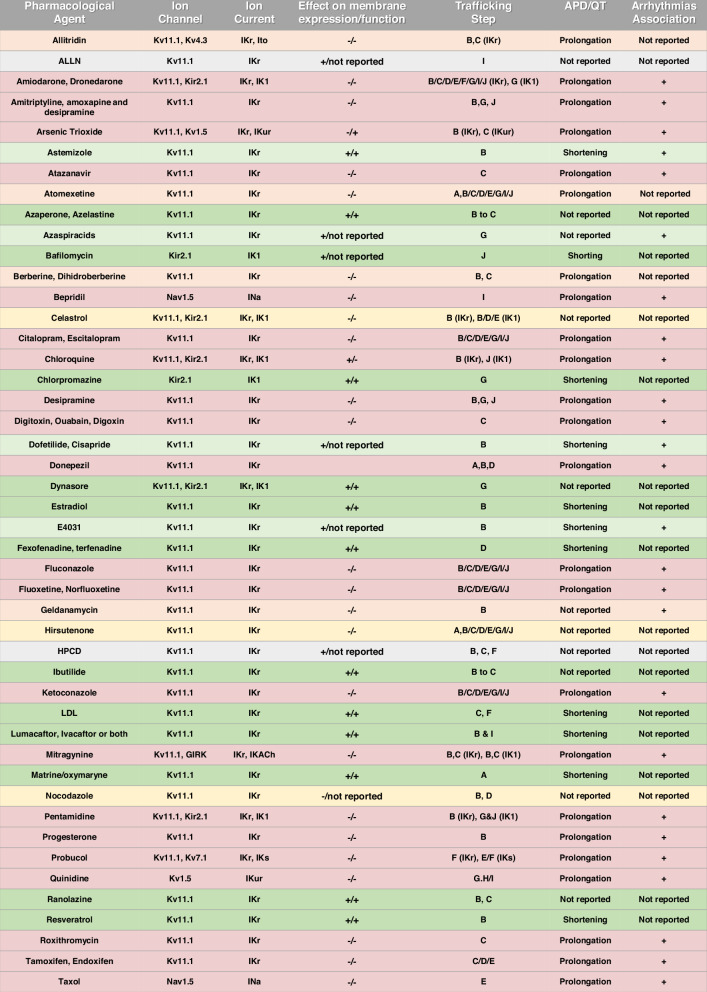
Pharmacological agents that cause cardiac ion channel trafficking defects or rescue ion channel trafficking defects associated with aLQTS or cLQTS.

Drugs that have been demonstrated to cause channel trafficking defects and APD and/or QT prolongation are marked in light red and those that also induce arrhythmias are marked in red. Drugs that disturb ion channel trafficking but have not been reported to cause APD/QT prolongation or association with arrhythmias are highlighted in yellow. In contrast, drugs that can rescue trafficking defects under pathological conditions (cLQTS or aLQTS) and have not been associated with proarrhythmic events are highlighted in green. Although some of them can improve the membrane expression of LQTS-mutated channels, others have also been associated with arrhythmia (light green), which does not qualify them as good therapeutic tools. Drugs that can rescue the membrane expression but their effects on current, APD, or QT interval are unknown (gray) could be potential pharmaceutical candidates.

Trafficking defects, QT and/or APD prolongation and association with arrhythmias.

Trafficking defects, QT and/or APD prolongation but not association with arrhythmias.

Trafficking defects, but not been associated with QT/APD prolongation and arrhythmias.

Rescue at protein level, but unknow functional effect on the APD or QT.

Rescue, shorten QT and/or APD but associated with arrhythmias.

Rescue, shorten QT and/or APD and not associated with arrhythmias.
- Drugs affecting lipid rafts and caveolae: The disruption of intracellular cholesterol homeostasis can lead to alterations in cellular membrane composition^133,193,209^. For example, progesterone impairs hERG trafficking by disrupting intracellular cholesterol homeostasis^210^. Probucol (Table 2), a cholesterol-lowering drug that causes LQTS, reduces I_Ks_ and I_Kr_ by decreasing plasma-membrane protein expression, and the addition of LDL almost completely restored probucol-reduced hERG membrane expression^193^. hERG trafficking defects induced by disrupted cholesterol homeostasis can also be corrected by the cholesterol-redistributing or a sterol-binding agent HPCD (2-Hydroxypropyl-β-cyclodextrin)^210^ .
- Targeting the forward or backward trafficking machinery: Trafficking-disrupting drugs can now be divided into those that interfere with forward trafficking and those that interfere with backward trafficking, although some drugs affect both pathways. Drugs may interfere with channel trafficking at several points of its life cycle, such as by inhibiting folding in the ER, enhancing proteasome degradation, altering channel glycosylation, inhibiting endocytosis, affecting recycling, lysosomal degradation, disturbing cholesterol homeostasis, inhibiting calmodulin signaling or enhancing microtubule polymerization^211^. Typically, the effects of a drug on channel endocytosis manifest in the “short-term” (30 min to 6 h) and become more significant with prolonged drug exposure (after 24 to 48 h^32^. In contrast, effects on backward trafficking have only been reported after “long-term” drug exposure (16 to 48 h)^191,199,212^. We carefully state “short- and long-term” because most of these results have been obtained using heterologous expression systems, and the therapeutic conclusions cannot be applied directly to the clinic. The mechanisms involved in forward trafficking after the gene transcription, as described previously in Fig. 2, are: [B] Protein folding and proper assembly; [C] Post-Translational modification & Glycosylation; [D] Anterograde vesicular trafficking along microtubule; and [E] Membrane insertion. Table 2 reflects different drugs that may influence one or more of these mechanisms simultaneously, which unleashes the complexity, compromising channel trafficking. For instance, estradiol increases membrane trafficking of Kv11.1 through a quantitatively greater association of the channel with chaperone complexes (Hsp90 and Hsp70) and enhancing Kv11.1 protein folding and assembly; however, estradiol failed to restore membrane trafficking of a mutant KCNH2 channels^103^. The cardiac glycosides digotoxin, ouabain, and digoxin (Table 2) can alter hERG protein trafficking by altering the glycosylation^131,213,214^. Roxithromycin, an oral macrolide antibiotic agent that has been repeatedly reported to provoke excessive prolongation of the QT interval and *TdP*, inhibits the maturation of hERG channels, disrupting its membrane trafficking^204^. Several drugs such as azaperone, azaspiracids, azelastine, ibutilide, ranolazine, and resveratrol have been shown to rescue the membrane expression of Kv11.1 in pathological conditions by improving the transition from channel assembly and maturation by post-translational modification or glycosylation^215–217^. Nolodazole, tamoxifen and endoxifen (Table 2) are among the drugs that can impair the anterograde vesicular Kv11.1 trafficking along microtubule^218–220^. Fexofenadine and terfenadine can rescue anterograde vesicular trafficking-defective LQT2 variants, alleviating the LQTS phenotype^205^. A microtubule-stabilizing agent, enhanced tubulin polymerization by taxol has been reported to induce a 50% decrease in Na_v_1.5 current amplitude in cardiomyocytes evoking arrhythmias^221^. Several compounds have been demonstrated to successfully target the backward ion channel trafficking. For example, antidepressants such as amitriptyline, amoxapine, and desipramine exert multiple effects on hERG, particularly prone to degradation^199,222,223^. Chloroquine and the antiprotozoal pentamidine also inhibit lysosomal degradation of Kir2.1 channels precipitating LTA^32,207,224^. The antiarrhythmic drug, quinidine, stimulates rapid Kv1.5 internalization and its lysosomal degradation in atrial myocytes in a dose-; temperature- and calcium-dependent manner^201^. Some drugs have been shown to improve membrane expression mainly of Kir2.1 by targeting the retrograde vesicular trafficking (internalization) along microtubule; examples are chlorpromazine, bafilomycin A1 (Baf), chloroquine and dynasore^207^. By inhibition of proteasomal degradation using ALLN or Lumacaftor also showed to rescue the membrane expression of dysfunctional hERG channels in LQTS^225,226^.

- Alteration of cardiac ion channel gene expression: Trafficking defects may be worsened by drug effects on gene expression, especially after long-term (16 h to days) treatment^191^. Moreover, trafficking defects could be caused by modifying gene expression by altering the acetylation status of lysine residues of histone proteins; as has been demonstrated, histone deacetylase inhibitors can prolong cardiac repolarization through transcriptional mechanisms^227^. Certain compounds collected in Table 2 show a potential inhibition of hERG gene expression, although the exact mechanisms are unknown. For instance, atomoxetine and hirsutenone (Table 2) have been shown to inhibit the *KCNH2* gene at higher concentrations but already affect Kv11.1 maturation at lower drug levels^228,229^. Donepezil, an acetylcholinesterase inhibitor, inhibits Kv11.1 channel expression and trafficking to the plasma membrane^230^. On the other hand, long-term treatment with marine and oxymatrine (Table 2) was described as the up-regulation of hERG at both mRNA and protein levels via an increase in the expression of transcription factor Sp1, an established transactivator of the *KCNH2* gene^231^.

We propose an update of all the drugs described so far that have the potential to alter cardiac ion channel trafficking (Table 2). Table 2 illustrates which ion channels are affected, their mechanism, their effect on the APD or QT interval, and their association with arrhythmias. It reveals that 25% of the channel trafficking modulator drugs known so far can rescue trafficking defects in cLQTS variants and drug-induced LQTS, supposing a promising strategy. This scenario supports the use of drugs to correct or rescue channel trafficking defects.

Novel types of therapies are also being contemplated to correct defective ion channel trafficking. For example, targeting deubiquitination using engineered deubiquitinases (enDUBs) can rescue the functional expression of disparate mutant ion channels underlying LQTS^232^. Another ingenious study using designer receptors exclusively activated by designer drugs (DREADDs) showed that β-arrestin signaling plays a role in hERG regulation^233^. It was found that by activating exclusively β-arrestin-biased M3 muscarinic receptor-based DREADD (M3D-arr) using the otherwise inert compound clozapine-N-oxide, M3D-arr activation increased mature hERG expression and current^233^. M3D-arr recruited β-arrestin-1 to the plasma membrane and promoted phosphoinositide 3-kinase-dependent activation of protein kinase B (Akt). The activated Akt acted through phosphatidylinositol 3-phosphate 5-kinase and Rab11 to facilitate hERG recycling to the plasma membrane^233^.

Among other new promising strategies, we find different gene therapies. Gene replacement therapy, particularly through overexpression of therapeutic genes, has been a promising approach in treating genetic disorders caused by disease variants that generate mutant proteins^234^. However, this strategy presents a complex landscape regarding its potential therapeutic benefits and risks. It has been shown that overexpression of therapeutic genes could be helpful in restoring function when the mutant protein is dysfunctional or absent^235^, for correction of loss-of-function mutations^236,237^ or to compensate for insufficient protein levels^238,239^. However, the potential toxicity and risk associated with gene therapy used for heritable arrhythmia must be carefully considered. For example, overexpression of proteins can lead to an increased risk of protein misfolding and aggregation, which can be toxic to cells and arrhythmogenic. This is particularly relevant for channelopathies, where the folding and dimer/tetramers formation of α-subunit is a key process for ion channel trafficking and could exacerbate the formation of toxic aggregates^240,241^. The overexpression of a gene may also disrupt normal regulatory mechanisms and homeostasis within the cell^242^. Importantly, in cases where the mutant protein exerts a dominant negative effect, simply adding a functional gene may not be sufficient. The mutant protein could interfere with the normal function, potentially exacerbating the disease. For example, integrin-linked kinase (ILK) is crucial for cardiomyocyte survival and function^243^. White DE et al. showed that dominant-negative mutations in ILK led to impaired heart function, dilated cardiomyopathy, and heart failure^243^. The overexpression of normal ILK was not sufficient to rescue the phenotype caused by the dominant-negative mutations, indicating that the mutant ILK proteins interfered with the normal ILK function, leading to detrimental effects on heart structure and function^243^.

To apply the gene therapy approach for treating LQTS, the adeno-associated virus (AVV)-mediated introduction of the WT version of genes encoding a cardiac ion channel or its regulatory subunits could be a promising option. Many experts consider AAVs to possess several advantages over other viral and non-viral methods for gene transfer, including high safety, low immunogenicity, sustainable and stable exogenous gene expression, etc., which makes AAV one of the most promising candidates for the treatment of many human genetic disorders and hereditary diseases^244,245^. Therefore, AAV technology has been approved for clinical use in humans. There are now five treatments approved and currently available for commercialization, i.e., Luxturna, Zolgensma, the two chimeric antigen receptor T cell (CAR-T) therapies (Yescarta and Kymriah), and Strimvelis (the gammaretrovirus approved for adenosine deaminase-severe combined immunodeficiency (ADA-SCID) in Europe). Many other treatments are undergoing clinical trials. Moreover, toxicity has been described in rare cases when high viral doses are applied or in the liver by a long-term clonal expansion of cells carrying the transgene^246^.

It is necessary to modify the viral capsids of AAVs to enhance their tissue-specific tropism while reducing off-target effects in other tissues^247^. This approach is critical for gene therapy that aim to treat heart-related diseases. The use of AAV serotype 9 (AAV9) in mouse models has demonstrated cardiac tropism^248–250^, high infection rates (over 95%), and remarkable genetic loads of AAV9 vectors delivered to the heart (over 60% of the cardiomyocytes having 1–3 copies of the transgene)^251–253^. The system always generates a mosaic-like cellular distribution of the protein of interest in the heart. Nevertheless, western blotting experiments have demonstrated that protein levels in uninfected and AAV9-infected mice were not significantly different^251–253^. Moreover, AAVs do not integrate into the host genomes and will eventually be diluted as the cell undergoes repeated replication^254^. However, as the cardiomyocytes are quiescent cells, researchers can take advantage of the non-integrative properties of AAVs without suffering from the inconvenience of viral load dilution.

The channel or desired protein needs to be small enough to be contained in the AAV capsid. The AAV vectors have a limited packaging capacity: up to 4.8–5.2 kb for efficient packaging, and it includes the gene and its non-coding regulatory sequences (such as inverted terminal repeats, promoters, enhancers, internal ribosome entry sites, and poly-A tails)^255^. Among the disadvantages of using AAVs are their inability to target specific tissues effectively, the existence of neutralizing antibodies, the wrong molecular targets, and inadequate administration doses, which need to be considered. However, many laboratories worldwide have described that cardiac-specific expression of transgenes by AAV technology is not proarrhythmic per se. The invaluable utility of this tool has been demonstrated for treating arrhythmogenic right ventricular cardiomyopathy^256^. In addition, AAVs have been successfully used for cardiac gene therapy in different models, like Brugada Syndrome and CPVT by gene complementation or gene editing^257–259^.

Another variant of gene therapy, suppression-and-replacement gene therapy, has been applied in human iPSC-derived cardiomyocytes (hiPSC-CMs) from LQTS1 patients^260^. In that recent study, the authors achieved complete correction of LQTS1 suppressing and replacing *KCNQ1* to normal WT levels, consequently leading to AP shortening. iPSC technology would be described in detail in a subsequent section. Yet another innovative and remarkably interesting genetic option for cardiac diseases is the use of antisense oligonucleotide (ASO) or small interfering RNAs (siRNAs) therapy^261–264^. ASOs or siRNAs, short single- or double-stranded oligonucleotides respectively, can bind to target RNAs, activate cytoplasmic degradation of target RNAs or modulate splicing of pre-messenger RNAs inside the nucleus^265^. ASO therapy has been recently applied to the treatment of Timothy syndrome type 1. The authors have reverted the G406R variant in Ca_V_1.2 by switching the splicing of the mRNA exons; instead of including the exon 8A of Ca_V_1.2, where the nucleotide change is, they have used ASO for increasing the presence of the non-mutated exon 8^266^. An emerging area that has gained recognition in recent years is the use of microRNAs (miRNAs or miRs) as therapy or accessible biomarkers of multiple heart diseases^267–269^. siRNAs and miRNAs have many similarities, as both are short double-stranded RNA molecules that silence genes at the post-transcriptional level by targeting mRNA^270^. However, the primary distinction between them lies in their specificity: siRNAs are highly specific, typically targeting a single mRNA, while miRNAs can regulate multiple mRNAs binding to complementary sequences in the 3’ untranslated region (3’ UTR)^271^. They also differ notably in their origins, processing pathways, mechanisms of target recognition, modes of action, and functional roles within the cell^270^. miRNAs can be used to modulate the duration of the AP, by directly targeting ion channel expression and secondarily their trafficking and membrane expression. Diverse miRNAs have been associated with prolongation of the AP and, consequently, the QT interval. A study demonstrated that both the expression and consequently the function of the hERG channel can be regulated by miR-134, miR-103a-1, miR-143, and miR-3619^272^. Also, upregulation of miR-1, miR-133, miR-21, and miR-23a contributes to arsenic oxide (As_2_O_3_)-induced hERG deficiency and cardiac electrical remodeling^273,274^. Yet another recent study identified miR-365 as a primary miRNA to regulate AP repolarization by repressing its direct ion channel targets^275^. The inhibition of miR-365 appears to normalize the abnormally prolonged AP in LQTS hiPSC-CMs by targeting *KCNQ1*, *KCNH2*, *KCNJ2*, *CACNA1C*, *KCNC3*, *KCNA1*, and *KCNJ3*, the dominating ion channel isoforms that determine cardiac repolarization, offering this miR as a potential therapy for the treatment of LQTS^275^. Therefore, several studies have proposed RNA therapy using miRs or antagomiR to modulate the gene expression of cardiac ion channels as a promising tool in LQTS treatment. However, this emerging world of miRNAs should be taken with caution, as a single miRNA can influence unrelated proteins encoded by different genes with similar 3’ UTR sequences. So, the effects of disturbing miRNA copy numbers may be extensive and cause remarkable off-target effects^276,277^. On the other hand, the application of gene therapy to increase the expression of GTPases critical for LQTS-trafficking deficient variants would be a valuable strategy^111^. Thus, trafficking-deficient variants, not necessarily implying ion channel malfunctioning, could reach their destinations more effectively.

As Chidipi et al. demonstrated recently, peptide-based therapies can be a new option for the treatment of channelopathies. They designed a fusion protein between the Fc fragment of the human IgG1 and a peptidotoxin (tertiapinQ) to block, specifically, Kir3.1/3.4 channels responsible for I_KACh_^278^. The design of bioengineered peptides that can improve the movement of trafficking-deficient variants causing any subtype of LQTS would also be another invaluable tool for treating patients suffering from these syndromes.

With all, each therapeutic approach must be tailored to the specific genetic and molecular context of the disease to maximize benefits and minimize risks. Rigorous preclinical and clinical testing is essential to evaluate the safety and efficacy of gene replacement strategies.

### Membrane protein macromolecular complexes: *channelosomes* and *chansporters*

Protein behavior must be understood inside macromolecular complexes. Membrane proteins contain within their primary sequences information that determines which other proteins they can interact with to help them traffic to the cell surface, how long they remain there, and what their fate is once they are recycled from the plasma membrane. All such processes imply protein–protein interactions^279^. When two or more different membrane proteins co-assemble, they can influence each other’s function.

In the case of ion channels, they often incorporate into macromolecular complexes called “channelosomes”, which are highly organized macrostructures where channels interact with one another and with regulatory, adaptor, scaffold, and signaling proteins in an orchestrated fashion^251^. *Channelosomes* ensure suitable electrical signaling and coordination of heart contraction^183,280–283^. Their proper structural architecture and function are crucial for keeping a healthy heartbeat, and their disruption is intimately linked to the development of arrhythmias^284,285^. The high degree of structural and functional specialization in cardiomyocytes implies that the trafficking and orientation of cardiac ion channels follow distinctive and specific pathways^286^. Importantly, the intricate process of electrical activation through the myocardium is likely governed by the spatial, temporal, and differential distribution of *channelosomes* within specific microdomains in individual cardiomyocytes, establishing the anisotropic ratio of impulse propagation^287^. The mechanisms that regulate the targeting of ion channels to specific sarcolemma subdomains and their integration into macromolecular complexes have been poorly explored. Formation of macromolecular complexes is integral in various processes, including transcription, translation, oligomerization, trafficking, membrane retention, post-translational modification, turnover, function, and degradation of all known cardiac ion channels and their regulatory subunits^288^. Further, maintaining the precise timing required for each cardiac beat requires an analogous precision in the assembly and organization of sodium, calcium, and potassium channel complexes within specific subcellular microdomains, all influencing transcriptional strategies in cardiomyocytes. Physical proximity in these domains enables swift and efficient interactions^286,288^.

While ion channel proteins facilitate ion diffusion down an electrochemical gradient across the cell membrane, other membrane proteins, distinct from channels, transport ions across the cell membrane by facilitative diffusion. Even though membrane ion channels are known to cooperate functionally with transporters, recently they have been shown to interact physically^279^. These so-called channel-transporter complexes or “chansporters” represent a ubiquitous and evolutionarily conserved class of signaling complex that may serve to circumvent potentially large spatial signal integration barriers between channels and transporters, and the ions and other substrates they transport^279^. These structures are not only specific to heart tissue. For instance, Kv7.1 and KCNE2 form complexes with SMIT1, an active transporter that moves sodium down its electrochemical gradient to facilitate the uphill transport of Myo-inositol into cells. Such complexes contribute to regulating neuronal excitability^289^. Additional examples of *chansporters* have been found in many other organs, including the gut^290^, the brain^291^, the mammary glands^292^, and the kidney^293^.

Up to now, we have described the formation of membrane macrocomplexes of a deterministic nature emphasizing the highly spatial/temporal organized assembly of these complexes underlining a functional consequence. However, we should also consider the formation of membrane macrocomplexes using stochastic models. Stochastic models focus on the random and probabilistic nature of molecular interactions^294^. These models account for the dynamic and fluctuating nature of complex formation influenced by different factors^295^. Macroscopic properties emerge from the collective behavior of numerous simple stochastic events^296^. Models consider the temporal evolution of systems, accounting for the transient nature of many interactions^297^. These models can capture the variability and heterogeneity observed in biological systems. Stochastic models allow the inclusion of diverse sources of biological noise, such as variability in gene expression and environmental fluctuations^298^. They provide insight into how cells can adapt to changing conditions and maintain robust functions despite inherent noise^298^. In 2007, Thiem et al., proposed that protein cluster formation and growth is a stochastic self-assembly process in which newly synthesized proteins diffuse through the membrane and then join existing clusters or create new clusters anywhere^296^. Sato et al., endorsed this approach and proposed that their size is regulated by a common feedback mechanism^299^. The model’s three parameters represent channel cluster nucleation, growth, and removal probabilities estimated based on experimental measurements of different channels in various cell types^299^. Protein nucleation refers to the insertion of channels into the membrane. It can be random or favored by the up/down-regulation of a modulator^299^. After nucleation, there is a step of rapid cluster size growth in which other ion channels are inserted randomly closed to a nucleating channel until they reach a steady state. The steady-state form is maintained by a relatively fast turnover rate^299^. The rates of cluster nucleation, growth and degradation, all of them defining the mean lifetime of a channel at the membrane, differ between cells and differentiation/maturation stages^299^. For instance, the authors observed that Ca_v_1.2 channels in ventricular myocytes form clusters by stochastic self-assembly in which the cluster area follows a steady-state size distribution and constant densities^299,300^. The results suggested the existence of a regulatory feedback mechanism controlling channel cluster size and density at specific regions^299^. In this sense, trafficking impacts channel number and organization. Moreover, in the case of channels that undergo functional coupling, a higher cluster size enhances the coupling power, resulting in functional consequences (i.e., increase in current amplitudes).

Then, both deterministic and stochastic mechanisms recognize the importance of microdomains composed by specific sets of ion channels and related proteins in cell function. While the deterministic approach supports highly specialized clusters’ organization, Sato and colleagues consider that Ca_V_1.2 channel clusters are stochastically self-assembled operating within a restricted cellular domain, but not throughout the entire cell^299^. The stochastic model proposed by Sato et al. also considers three potential scenarios in which a change in a physiological process alters ion channel cluster area or density leading to pathological conditions^299^. First, they proposed that the insertion of channels at the plasma membrane could be enhanced by the up-regulation or activation of a signaling pathway. In this scenario, the increase in the expression of the channels will enhance the available channel pool that can be inserted at the plasma membrane, increasing cluster density^299^. Second, they also consider the possibility that the insertion of new channels is favored to occur in the same sites where other channels have been previously inserted because of the up-regulation of an interacting protein^299^. For example, cBIN plays a crucial role in organizing membrane microfolds within cardiac t-tubules and its expression has been related to increased Ca_V_1.2 channel expression at membrane^301–303^. According to the stochastic model, the membrane expression of BIN1 could function as an attractor to increase the local concentration of Cav1.2 channels at specific sites, enhancing their clustering^299^. Consistent with this, De La Mata et al. found that the overexpression of cBIN1 in human embryonic stem cell-derived cardiomyocytes (hESC-CMs) increased Ca_V_1.2 cluster size^304^. In a deterministic situation, the clustering phenomenon may be explained by the fact that cBIN acts anchoring microtubules and helping the organized trafficking machinery where newly synthesized Ca_V_1.2 channels are delivered to the cell surface. The last scenario, the removal of individual ion channels or ion channel clusters from the plasma membrane is enhanced by an interacting protein^299^. In this scenario, the activation of these proteins will likely be associated with an overall decrease in channel membrane dwell time and a decrease in cluster size^299^.

Both deterministic and stochastic points of view contemplate that the membrane expression of ion channels involves a feedback mechanism necessary to reach and maintain a steady state^299^. This mechanism includes a multitude of processes necessary to achieve a precise spatial and temporal localization committed to a functional purpose. We can simplistically assume that the number of ion channels in the membrane can be referred to as the difference between their rate of repositioning (by anterograde trafficking) and disappearance (by retrograde trafficking). An alteration in any of these rates will result in a new equilibrium, determined by an altered amount of membrane ion channel relative to the physiological condition. For example, the trafficking-deficient Kir2.1^Δ314-315^ variant linked to ATS1 produces a reduction in the amount of channel reaching the plasma membrane^251^. Although compensatory mechanisms may exist at the cellular level, in cells expressing that variant this effect is not compensated by a reduced Kir2.1^WT^-retrograde traffic that allows maintaining an adequate amount of ion channels in the membrane^251^. Therefore, we speculate that new steady states in terms of cluster’s number and density are reached under pathological conditions. This opens the possibility of interesting insights, but they need to be demonstrated. For example, treatment of an anterograde trafficking-deficient variant with a retrograde trafficking inhibitor could theoretically rescue the amount of channels at the membrane. However, the steady state for each cluster containing ion channels could be slightly different even between cells expressing the same variant, contributing to the arrhythmogenic phenotype and its variable expressivity. This effect has been observed in studies of trafficking-deficient hERG channel variants, with the mutant versions showing remarkable variability in terms of membrane expression relative to the wildtype condition^305^. However, to what extent this variability responds to experimental or technical issues or is directly due to a variant-mediated mechanism remains unanswered. In general, most scientists attribute the condition of trafficking-deficient variants directly to the malignancy, while intercellular variability in terms of newly reached steady states remains in the background. Future studies could demonstrate whether these mechanisms contribute to facilitate the creation of an arrhythmogenic substrate. Likewise, a classification of trafficking-deficient variants that considers their intercellular variability and malignancy could also help resolve this question.

Stochastic models have limitations such as they rely exclusively on channel number and do not consider the state of the channel (e.g., open, deactivated, inactivated, or desensitized). Thus, a desensitized or inactivated channel would have the same probability of being removed by internalization or endocytosis than a deactivated or open channel, and this probably is not the case. They also fail to explain whether membrane channels are internalized individually or in clusters. Additionally, it remains untested whether channel membrane dwell time is higher in rapidly dividing cells compared to nondividing cells like ventricular myocytes, and how disease states might affect channel dynamics. In conclusion, stochastic models do not contradict that precise organization is crucial for health, but rather provide a framework for understanding how cells can maintain their function despite inherent noise and variability, offering insights into how cells can adapt to perturbations. Stochastic models offer a complementary perspective and the integration of deterministic and stochastic approaches could improve our understanding of the intricate and robust nature of the cellular machinery.

### Importance of *channelosomes* in channel trafficking associated to LQTS and SCD

The simplest and oldest known *channelosomes* involved in ventricular function are formed by different families and subfamilies of voltage-gated cardiac ion channels and their respective ancillary β-subunits^160,306,307^. Kvβ subunits influence trafficking, stability, and functional and pharmacological properties of the overall channel complex^306,308–311^. By far the most studied transmembrane Kv ancillary subunits are the KCNE subunits, also called MinK-related proteins (MiRPs), essential for ventricular repolarization in some cases because their variants are associated with human LQTS^160,279,306^, as discussed above. MinK (encoded by *KCNE1*) associates with the Kv7.1 α subunit in the ventricles of mammals including Guinea pigs, horses, and human^153,312,313^. MiRP1 (encoded by *KCNE2*) regulates the hERG channel and loss-of-function MiRP1 variants are associated with cLQTS^41,314^. However, MiRPs’ promiscuity probably renders the actual situation more complex because, among other things, MinK can also regulate hERG, and other MiRPs can regulate Kv7.1, so variants in their encoding genes can result in a range of phenotypes^152^.

In the heart, α-subunits of Na_V_1.5 assemble resulting in coupled gating properties^315^, and Ca_V_1.2 channels amplify e-c coupling by forming clusters of two to five channels to enhance calcium influx^300^. The gain-of-function variant G406R in Ca_V_1.2 linked to Timothy Syndrome type 1 alters the activation of calcium/calmodulin-dependent protein kinase II (CaMKII) and contributes to a reduction in I_Na_ density, creating a favorable substrate for the onset and maintenance of ventricular arrhythmias^316^. Hence, these channels are indirectly connected and reciprocally regulated. Recent in-vivo mouse studies on the arrhythmogenic cost of trafficking-deficient Kir2.1 variants in ATS1 have demonstrated the crucial importance of the interplay between Kir2.1 and Na_V_1.5 to normal cardiac electrical function^251^. In addition to reduced I_K1_, ATS1 patients also have reduced I_Na_ due to disruption of the Kir2.1-Na_V_1.5 *channelosome*^317^. The functional consequences are manifold: the resting membrane potential is depolarized, excitability is reduced, AP upstroke velocity is also reduced, and APD and QT interval are prolonged^251^. Kir2.1 and Na_V_1.5 associate early in their respective biosynthetic pathways and share common forward trafficking mechanisms as they form the Na_V_1.5-Kir2.1 *channelosome* and anchor to the cell membrane^182,251,317,318^. Cardiomyocytes express several membrane-associated guanylate kinase (MAGUK) superfamily proteins involved in cardiac ion channel trafficking, scaffolding and function^286,319,320^. MAGUK proteins are characterized by several protein–protein interaction domains within their primary sequences, explaining how channel complexes remain anchored to the plasma membrane^319,321–325^. Synapse-associated protein 97 (SAP97), the most thoroughly characterized MAGUK protein in cardiomyocytes, is involved in the scaffolding machinery^286^. It anchors several cardiac ion channels to the plasma membrane microdomains and contributes to formation of macromolecular complexes involving different ion channel families^51,286,321^. SAP97 coordinates the assembly of Na_V_1.5-Kir2.1 *channelosomes* at the T-tubules and intercalated discs, modulating their respective currents, I_Na_ and I_K1_, which play essential roles in cardiac electrical excitation and impulse propagation. The distinctive arrangement of the Na_V_1.5-Kir2.1 *channelosome* enables mutual positive modulation; i.e., when I_K1_ increases, I_Na_ also increases and vice versa^285^. Similarly, an increase in Na_V_1.5 expression at the membrane correlates with a decrease in the Kir2.1 internalization possibly involving retrograde trafficking^285^.

Kv4 channels also form a tripartite complex with SAP97 and CaMKII in cardiac myocytes regulating this ion channel surface expression and CaMKII-dependent regulation^322^. While no SAP97 variants leading to LQTS have been described, recently it was shown that the common p.P888L SAP97 polymorphism increases the fast component of the transient outward current (I_to,f_), and abbreviates the APD and the QT interval in mice. The consequences appeared mediated via a CaMKII-dependent effect that may increase the risk of arrhythmias^326^. Similarly, cardiac-specific ablation of SAP97 in a murine model demonstrated ECG abnormalities, an AP prolongation, and arrhythmias^327^. More recently, a new heterozygous SAP97 variant (p.R519H) in exon 15 of the *DLG1* gene was identified in a large family. Six out of 12 members were carriers of the variant and all six had a Brugada phenotype^328^. Another recently described cardiac Na_V_1.5 partner is the MAGUK protein CASK, which co-localizes at the lateral membrane with syntrophin and the dystrophin protein complex and negatively regulates I_Na_ by impeding Na_V_1.5 anterograde trafficking^319^. In contrast, α1-syntrophin forms PDZ-binding domain-mediated *channelosomes* with both Na_V_1.5 and Kir2.1 at the lateral membrane of the cardiomyocyte^182,183,327^. A recent study in hiPSC-CMs reprogrammed from skin fibroblasts of Duchenne muscular dystrophy patients with cardiomyopathy had a Na_V_1.5–Kir2.1 *channelosome* dysfunction, which could be rescued by α1-syntrophin to restore excitability and prevent arrhythmias^183^. Moreover, the close interaction of α1-syntrophin with the Na_V_1.5–Kir2.1 *channelosome* has been further proven by the demonstration that binding of a fragment of the Na_V_1.5 N-terminal domain to α1-syntrophin increases membrane density of human Kir2.1, Kir2.2 and Na_V_1.5 channels^182,317^.

Other cardiac ion channels have been shown to interact and form complexes. For example, Kv7.1 functions as a chaperone for hERG trafficking. The trafficking-deficient LQTS variant Kv7.1-T587M fails to show the chaperoning function that enhances hERG membrane localization with Kv7.1-WT, which explains the malignant clinical phenotype in affected patients^35^. Another study demonstrated that co-expression of Kv7.1 with hERG slowed the internalization of mature hERG and contributed to hERG membrane stability^329^. Several variants in CaM encoding genes are associated with LQTS as mentioned above. CaM complexes regulate the trafficking of diverse ion channels. For example, CaM is considered an obligate subunit of Kv7.1, modulating tetramer assembly, channel folding, and membrane trafficking^330^. Ankyrin-B is a critical multi-functional cardiac regulatory protein with key roles in ion channel trafficking, and membrane targeting, anchoring, and modulation^151^. Variants in the gene coding ankyrin-B are associated with multichannel dysfunction and LQTS4 (ankyrin-B syndrome)^40,151^. Another anchoring protein is AKAP9, whose defects have been identified in patients with autosomal dominant LQTS11^39^. AKAP9 regulates Kv7.1 trafficking, so variants in AKAP9 affect its interaction with Kv7.1 decreasing its trafficking and function^331^.

In the foregoing section, we have briefly described the macromolecular complexes formed by ion channels and other proteins that have been linked to altered ion channel trafficking in the context of the LQTS subtypes. Unraveling the intricacies of *channelosomes* in the context of LQTS holds promise for advancing our understanding of the disorder, identifying novel therapeutic targets, predicting drug interactions, and improving the management and treatment of affected individuals. However, these macromolecular interactions do not occur only in trafficking vesicles and at the plasma membrane. Recent evidence has revealed their importance in channel biosynthesis. Eichel EA et al. demonstrated that *SCN5A* and *KCNH2* mRNA transcripts, encoding Na_V_1.5 and hERG channels responsible for I_Na_ and I_Kr_ respectively, are part of a discrete “microtranslatome” during protein translation^332^. Both transcripts are regulated in a way that alters the functional expression of both channels at the membrane^332^. More recently, Jameson et al. proposed that ion channels controlling APD in ventricular cardiomyocytes co-synthesized from heterotypic pairs of *KCNH2, SCN5A, CACNA1C, and KCNQ1* mRNAs, conferring electrical stability by co-regulating complementary ion channels. Depletion of one mRNA results in a corresponding reduction in the partner mRNA. The functional consequence of this co-regulation is thought to be a buffering or compensatory effect that may mitigate changes that would otherwise trigger LTA^333^.

As the understanding of ion channels inside macromolecular complexes and their role in cardiac electrical function increases, it is important to highlight that the misfunctioning of a protein in such macrostructures can alter the behavior of many interactors that regulate cell excitability^288^. Variants in the genes coding for 6 of Na_V_1.5 interactors were reported in patients with altered electrical function that may lead to SCD^334^. Several variants affecting α1-syntrophin are described in families with LQTS in which some members have suffered SCD^185,186^. Genetic alterations in Cav3 have also been identified in patients with congenital LQTS and have demonstrated to increase I_Na_ and led to SCD^18,175^. Other variants affecting Cav3 have shown to reduce Kir2.1 membrane expression and, consequently, I_K1_^175^. Hence, they contribute to delayed repolarization, arrhythmia generation and SCD in Cav3-mediated LQT9^335^. On the other hand, Organ-Darling LE et al. demonstrated that hERG and KCNQ1, responsible for I_Kr_ and I_Ks_ respectively, present direct intermolecular interactions mediated by their COOH-termini^336^. Consequently, loss-of-function variants affecting these proteins would alter functioning of the delayed rectifying potassium currents and predispose to SCD^27^.

Macromolecular complexes play crucial roles in a wide collection of cellular tasks. Understanding their structure and composition will enhance the knowledge about heart functioning in health and disease. Meeting the aim of knowing the components of these macromolecular assemblies is key to identifying novel therapeutic targets to reduce the incidence of SCD, the leading cause of mortality that accounts for approximately 50% of all cardiovascular deaths and for 20% of total mortality in the industrialized world^9^. The precise percentage of LQTS trafficking-deficient variants directly leading to SCD is currently unknown, hence their stratification will help reduce the rate of SCD in LQTS patients^9^.

### Uncharted routes: unmasking the molecular culprits by interactome mapping

Proteins seldom operate independently; typically, a diseased condition is linked to an augmented or decreased group of signaling proteins, metabolic factors, and altered gene expression^337^. The term “interactome” has been defined as the complete set of biological networks wherein molecular interactions of a particular protein are functionally mapped^338^. Although interactomes usually imply protein–protein interactions, they may also refer to metabolic networks or gene regulatory networks^339,340^. Comparisons of interactomes among different species can also determine the degree of conservation or dissimilarity among the networks involved in health and diseases^341,342^. Interactome studies contribute to a systems biology understanding of cardiac diseases^338^. These approaches consider the interconnectivity of molecular components and their dynamic interactions, leading to a more comprehensive view of the biological processes involved in diseases and helping identify which molecules or sets of molecules need to be targeted to control or cure a disease^338–340^. The study of interactomes has also made it possible to identify novel disease-causing variants, and by considering the broader network of molecular interactions, researchers can develop more accurate models for predicting disease risk and stratifying patients based on their susceptibility to cardiac events^340,343,344^. Interactomes have also been used to characterize the functions of unknown proteins. Usually, proteins that interact together are also likely involved in similar or the same biological processes^345^. Therefore, finding the interaction partners of an unknown entity can give clues about its function. Similarly, defining where a protein is localized can help understand how it works^346,347^. In general, interacting proteins tend to have the same or similar subcellular locations. Nevertheless, given the multiple processes a membrane protein needs to undergo throughout its lifetime, from translation and transcription, all the way to trafficking and membrane localization and degradation, the interactome of a given membrane protein may involve hundreds of varied interactors^339,340,348,349^. Moreover, given the structural complexity of cardiomyocytes, depending on their final microdomain (ICD, lateral membrane, T-tubule, organelle compartments) ion channels may have different interactors^93,341,350^. Thus, protein-protein interactions may enable inferences on the specific location of a protein.

Drugs usually interact with various molecular targets to produce their effects. While ideally, the primary interactions of a given drug are with specific targets, it may also bind to other ‘off-target’ proteins, which can be predicted by building the chemical-protein interactome of that drug^338^. Knowledge of interactomes allows exploration of network pharmacology, where drugs are designed to target specific nodes within the interactome^351^. Interactome mapping can also contribute to personalized medicine by helping identify the interactions that contribute to a disease phenotype and reveal novel therapeutic targets and the repurposing of existing drugs for treating LQTS, Brugada syndrome, and other diseases^340,349,352^.

Ion channels have been extensively characterized in heterologous expression systems. However, their behavior in-vivo often deviates from expectations due to complex interactions with various signaling/scaffolding proteins^353^. Such interactions strongly influence their trafficking, localization and function, interfering with their connection to intracellular second messengers and other signaling pathways^287,354,355^. In addition, ion channels have functions that extend beyond their conventional roles in ion conduction^353^. Therefore, ion channels must be approached not only as dynamic membrane ion filters but also as signaling complexes^353^, reinforcing the importance of knowing their interactome. Research in this area is likely to provide information on the role of interactors in the accessory functions of ion channels.

Recently, Maurya and colleagues outlined the ensemble of protein interactors for the 13 types of murine cardiac ion channels^350^. They reconstructed human cardiomyocyte ion channel networks from deep proteome mapping of human heart tissue and human cardiac-cell gene expression^350^. They demonstrated that 44% of the network proteins were significantly associated with an ECG phenotype. They identified and validated functionally 10 interactors, including two regulators of the Na^+^ current (epsin-2 and gelsolin)^350^. In the context of the LQTS, only a few studies have analyzed the interactome of ion channels or key proteins involved in the regulation of the QT interval. One such study aimed to elucidate the functions of the C-terminal domain of the LQT6-associated KCNE2 channel by identifying its protein interactors using a yeast two-hybrid analysis^356^. The authors identified Filamin C as a novel putative KCNE2 interactor under hypoxic conditions, which enhanced the understanding of ion channel function and regulation and also provided valuable information about possible pathways likely to be involved in LQTS pathogenesis^356^. Another recent study used proximity proteomics to generate a comprehensive map of the Kir2.1 interactome using the proximity-labeling approach BioID, which revealed as many as 218 high-confidence Kir2.1 channel interactions encompassing various molecular mechanisms^349^. The same study also explored the differences in the interactome profiles of Kir2.1^WT^ vs the Kir2.1 trafficking deficient variant Δ314–315^317,357^. The proteomic analysis helped uncover molecular mechanisms whose malfunctions may underlie ATS1 and other diseases like heart failure where Kir2.1 traffic and function may be altered. Functional studies that accompanied the interactome mapping approach also validated the functional relevance of PKP4 to I_K1_ modulation^349^. Mapping the Kir2.1 interactome provided a repository for numerous novel biological hypotheses on Kir2.1 and Kir2.1-associated diseases^349^.

The interactome of Na_V_1.5 has also been studied. LQTS3-linked and structure-guided variants in the Na_V_1.5 carboxy-terminus that disrupt its interaction with CaM cause a marked increase in the late Na^+^ current^358,359^. A recent study identified the role of CaM and the Na_V_1.5 interactome in regulating late Na^+^ current in mouse cardiomyocytes^360^. The study showed that endogenous fibroblast growth factor homologous factors (FHFs) may prevent late Na^+^ current. Hence, leveraging endogenous mechanisms may furnish an alternative avenue for developing novel pharmacology that selectively blunts late Na^+^ current in LQTS3^360^. Detailed analysis of the ion channel proteome/interactome provides fresh perspectives on the composition of ion channel complexes. It also illuminates how their dysregulation contributes to human disease^349,356,361,362^. Probing into less-explored or unknown pathways involved in the trafficking of ion channels within cardiomyocytes implies discovering hidden molecular elements that may contribute to irregular or disrupted ion channel function. Voltage Sensing domains (VSDs) are mobile specialized structures located at transmembrane segment domain S4 of voltage-dependent ion channels^363,364^. These specific domains detect changes, trigger conformational changes, and control the opening and closing of their structure^147,365^. VSDs are essential in cardiomyocyte excitability and physiological signaling^366–368^. VSDs also are targets for drugs and toxins that modulate ion channel activity^369–373^. Formerly it was thought that variants that affect amino acids located in the VSD of Kv7.1 channels only affect channel function^374^. Later, it was demonstrated that those variants also destabilize the structure, and disrupt trafficking and degradation of the Kv7.1 protein by the proteasome^154^. Folding-defective LQTS variants were observed to localize to the S0 helix of the VSD, where they interact with other elements^154^. Those observations revealed a critical role of the S0 helix as a central scaffold to help organize and stabilize the VSD of Kv7.1 and, most likely, the corresponding domain of many other ion channels^154^. Similarly, revealing new elements that alter ion channel trafficking in certain cardiac pathologies could benefit other diseases. For instance, the bridging integrator 1 (BIN1) is a membrane scaffolding protein that causes Ca_V_1.2 to traffic to T-tubules in healthy hearts and its reduction in heart failure impairs Ca_V_1.2 trafficking^375^.

In summary, mapping the interactomes of cardiac ion channels and other membrane proteins that govern the various phases of the cardiac AP is essential to continue advancing LQTS research and accelerate biomarker identification and therapeutic discovery.

### Modeling trafficking deficiency using LQTS patient-specific iPSCs

**Fig. 3:**
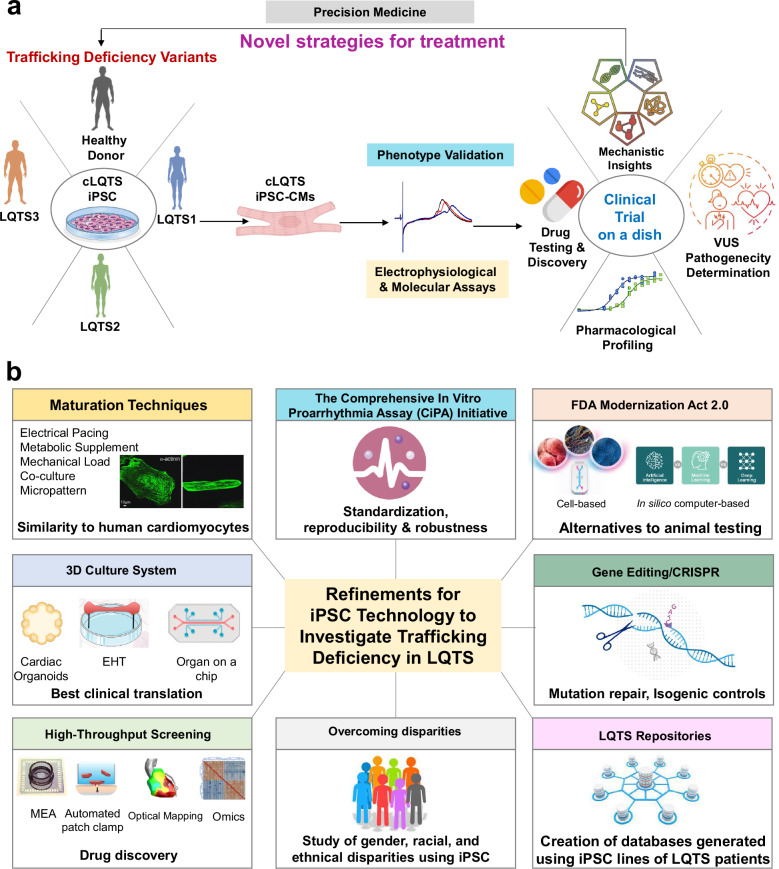
‘Channeling’ ion channel trafficking deficiency through iPSC technologies. **a** Precision Medicine for modeling ion channel trafficking deficiency in LQTS using iPSC technology. **b** Refinements for iPSC technology to investigate LQTS.

One of the significant breakthroughs in LQTS research and as promise of precision medicine is the ability to reprogram somatic cells from patients and healthy individuals into iPSCs and subsequently differentiate them into cardiomyocytes (hiPSC-CMs)^376,377^. Remarkably, iPSC technology is a powerful tool for in vitro human disease modeling and a highly relevant alternative to animal testing reducing the need for invasive procedures and supporting 3Rs principles^378^. Recruitment of patients with ion channel trafficking deficient variants is essential for advancing the understanding of LQTS pathology, and for developing personalized medicine approaches that can benefit those patients (Fig. 3a). LQTS patient-specific hiPSC-CMs constitute an unprecedented preclinical platform for developing the concept of “*clinical trials in a dish*”^376,377,379,380^. This human in vitro tool offers significant benefits for addressing mechanistic questions, pharmacological profiling, variants of uncertain significance (VUS), and drug discovery and testing (Fig. 3b)^330,379–381^. The effects of potential treatments can be assessed in LQTS patient-derived hiPSC-CMs, providing valuable insights into the most effective therapeutic strategies for that specific patient and encouraging personalized medicine^382–385^. Effective translation to clinical applications is essential for realizing their full potential in improving human health^386^. Ensuring that these models accurately predict human outcomes and translating iPSC-based discoveries into clinical trials involves significant ethical, regulatory and logistical hurdles on which further efforts should be invested^387,388^.

Several refinements to iPSC technology are being introduced that will benefit LQTS research (Fig. 3b). For example, the iPSC-CMs technology is evolving to build 3D models and engineered heart tissues (EHT), organ-on-chips and micro-physiological systems (MPS) to improve the maturation, replicate the heart tissue and LQTS complexity accurately (Fig. 3b). Recently, Veldhuizen J et al., developed a tissue-on-a-chip model of LQTS2 using a 3D co-culture of iPSC-CMs and cardiac fibroblasts (iPSC-CFs) carrying the hERG R531W variant within an organotypic microfluidic chip^389^. Disruption in hERG trafficking was observed in the hiPSC-CMs, providing insight into the potential mechanism underlying the pathophysiology of the R531W variant in LQTS2. Additionally, efforts were made to investigate the potential phenotypic rescue of LQTS2 on the chip using E-4031, ALLN (proteasome inhibitor), Thapsigargin (SERCA inhibitor), and nicorandil (I_KATP_ opener)^389^. Another advantage of iPSC technology is the ability to apply genetic interventions, such as CRISPR-Cas9 gene editing, to correct or introduce the genetic variants responsible for channelopathies^387,390–393^. One can combine hiPSC-CMs with gene editing techniques to systematically screen the impact of genetic LQTS variations on induced arrhythmogenesis. The latter may lead to the creation of more precise disease models, increase the understanding of individual susceptibility, and allow for the investigation of proarrhythmic risk in a patient-specific context^377^. CRISPR also allows the generation of isogenic controls for specific iPSC lines, which is of great relevance in this field^394^.

In parallel to iPSC-CMs development, the field of assessing the electrophysiological parameters is constantly changing and has been evolving with emerging methodologies and technologies to meet new challenges and needs. The priority is now being given to the HTS technologies using automated electrophysiology platforms which can rapidly screen a large number of conditions or drugs on different ion channel functions employing automated systems^395–397^. In addition, techniques to assess electrophysiological function in 3D culture systems are being developed which should provide more relevant insights than traditional cell culture models.

Using iPSC-CMs to model trafficking deficiency in LQTS involves recreating the cellular manifestations of the disease to study and better understand mechanisms. Specifically, how LQTS variants impact trafficking, function and the interactome of ion channels in cardiac cells. This approach should allow for personalized insights into the disease and hopefully more effective therapeutic strategies. In summary, we highlight iPSC-CM models to characterize fundamental trafficking defect mechanisms associated with LQTS. Next-generation treatments including small molecules, toxins, engineered peptides, gene therapy, miRNAs, and others will reap the benefits from the continuing development of the LQTS patient-specific iPSC-CM platform. State-of-the-art technologies including single-cell genome sequencing, proximity proteomics, CRISPR genome editing, and machine learning should bolster the approach. Deep phenotyping and HTS drug testing using LQTS patient-specific cardiomyocytes herald the upcoming precision medicine in LQTS (Fig. 3b). Furthermore, compared to some animal models (which lack some of the human ion channels, i.e., Kv7.1 in mice) and heterologous expression systems (which lack most of the cardiac complexity and trafficking machinery), hiPSC-CMs offer a much more reliable system for the study of LQTS. For example, many variants associated with LQTS may have an unknown effect on protein trafficking. However, analyzing the underlying mechanisms of the LQTS variants in overexpressing cell lines, characterized by the absence of potential LQTS-related protein interactors, limits the impact of results and hinders their application in clinical practice. Therefore, the use of hiPSC-CMs could reveal an even more relevant role of trafficking in the etiology of LQTS.

Despite the strengths and promise of iPSC technology, its major limitations for LQTS research must also be discussed. One of them is the incompleteness of disease modeling, since, although iPSCs can model many aspects of human diseases, they may not fully reproduce the complexity of human tissues and organs. This limitation may make it difficult to translate findings from iPSC models to actual clinical treatments. In addition, iPSCs may exhibit genetic and epigenetic variations that differ from the original donor cells^398,399^. These variations may affect the reliability and reproducibility of experimental results and therapeutic applications. The efficiency, reproducibility and purity of differentiation is also of great concern for the scientific community. Achieving efficient and accurate differentiation of iPSCs into specific cardiac cell types remains a challenge. Although iPSCs are derived from the patient’s own cells, the processes of reprogramming and differentiation can induce changes, and contamination by other cell types can affect efficacy. One significant limitation of using iPSC-CMs to study arrhythmias is their electrophysiological immaturity compared to native adult human cardiomyocytes^396^. iPSC-CMs often display functional characteristics more similar to fetal human cardiomyocytes, such as spontaneous beating, which might not fully replicate the complex electrical behavior of mature cardiac cells^396,400,401^. This immaturity can lead to discrepancies in electrophysiological behaviors and drug responses, potentially resulting in inaccurate predictions or conclusions^396^. Efforts are underway to improve the maturation strategies of iPSC-CMs to better resemble adult cardiomyocytes, but this limitation remains a challenge^402–404^. Standardization of protocols for generation, cardiac differentiation, maturation and quality control of iPSCs is essential for their widespread use in precision medicine.

In conclusion, iPSCs hold significant promise for advancing precision medicine through personalized disease modeling, drug discovery, and regenerative therapies in LQTS. However, addressing the limitations and ensuring effective translation to clinical applications is essential for realizing their full potential in improving human health.

## Conclusions and future perspectives

We have provided an overview of the diverse molecular players involved in LQTS-related ion channel trafficking that are known to date. We have also discussed the complex mechanisms that link a given variant with arrhythmia development in LQTS and other monogenic diseases. However, many other factors have yet to be discovered and explored. Genetically, most of the different types of LQTS have multiple variants associated with trafficking-deficient ion channels, particularly variants in hERG causing LQTS2. We emphasize the intricate processes involved in ion channel trafficking in cardiomyocytes and the importance of channelosomes and macromolecular complexes maintaining the balance for normal electrical function. Advancements in understanding ion channel interactomes are needed to identify novel therapeutic targets, predict drug interactions, and enhance the overall management and treatment of LQTS patients. Recognizing ion channels as signaling complexes and exploring the intricacies of their interactions with other proteins should open previously unexplored avenues for the understanding of LQTS. Such new avenues should also enable the designing of pharmacological tools that target macromolecular complexes instead of isolated channels. The integration of interactome studies, validation of functional relevance, and leveraging of endogenous mechanisms could lead to advancements in biomarker identification and targeted therapeutic strategies for LQTS.

As we move into an era of precision medicine, deservedly so thanks to new technologies, it is time to rethink the architecture of LQTS. Nowadays ascertaining the underlying causes of trafficking deficiency in LQTS patients is becoming increasingly feasible. iPSC-CM technology has revolutionized LQTS research, providing an advanced platform for disease modeling, drug testing, and understanding the molecular basis of LQTS.
